# Supplementary material for: Catastrophic impact of extreme 2019 Indonesian peatland fires on urban air quality and health
Source: Commun Earth Environ. 2024 Nov 2;5(1):649. doi: 10.1038/s43247-024-01813-w (PMC11531407; doi:10.1038/s43247-024-01813-w)
Supplement: Supplementary file 2 — Supplementary Information [file 43247_2024_1813_MOESM2_ESM.pdf]

Supplementary Information

Catastrophic impact of extreme 2019 Indonesian peatland fires on urban air quality and health

Grosvenor, Mark J.[1,2,3]* [https://orcid.org/0000-0003-0462-0461]; Ardiyani, Vissia[3,4,5,6] [https://orcid.org/0000-0002-0735-2952]; Wooster, Martin J.[1,2,3] [https://orcid.org/0000-0001-6375-7949]; Gillott, Stefan[4,5]; Green, David C.[4,5] [https://orcid.org/0000-0002-0695-4660]; Lestari, Puji[7] [https://orcid.org/0000-0001-6990-1331]; Suri, Wiranda[7] [https://orcid.org/0000-0002-2456-7365]

[1]Department of Geography, School of Global Affairs, King's College London, London, UK.

[2]NERC National Centre for Earth Observation, King's College London, London, UK.

[3]Leverhulme Centre for Wildfire, Society and Environment, King's College London, London UK.

[4]Environmental Research Group, Analytical & Environmental Sciences, King's College London, London UK.

[5]Environmental Research Group, School of Public Health, Imperial College London, London, UK.

[6]Nursing Department, Health Polytechnic of Palangka Raya, Palangka Raya, Indonesia

[7]Facaulty of Civil and Environmental Engineering, Institute of Technology, Bandung, Indonesia.

*Corresponding author: Mark Grosvenor (mark.grosvenor@kcl.ac.uk)

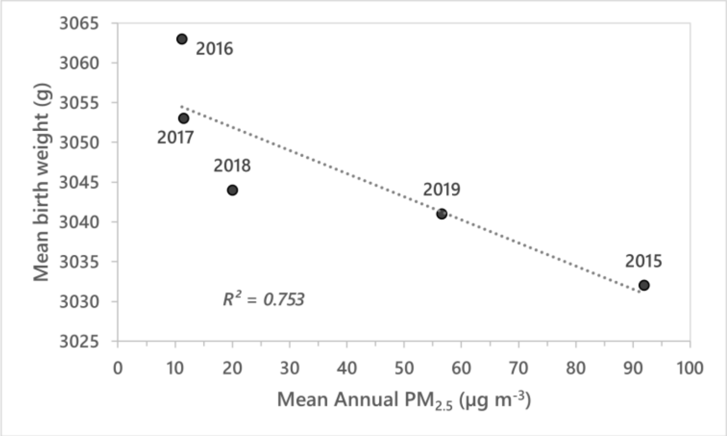

Supplementary Fig. 1 | Mean annual $PM_{2.5}$ concentrations and birthweights in Palangka Raya. Mean annual $PM_{2.5}$ concentrations are calculated from CAMS EAC4 reanalysis dataset[1] (as in Fig. 1). Mean annual birthweights are from one hospital (Doris Sylvanus) and ten midwifery clinic datasets comprising a total of 10815 birthweights between 2015 and 2019 (minimum 1464, maximum 3018 annual births).

Supplementary Notes 1: Comparison of $PM_{2.5}$ Concentrations and Satellite Active Fire Data

Fire Radiative Power (FRP) estimates from active fire (AF) pixels detected by the satellite-based Visible Infrared Imaging Radiometer Suite (VIIRS) every 12 hours (around 1pm and 1am local time) are shown in Fig. 2b and Supplementary Fig. 2 (see Schroeder et al.[2] for a description of VIIRS AF detection capabilities). These VIIRS

AF data have a spatial resolution of 375 m at nadir, and a very limited amount of off-nadir pixel size increase due to VIIRS design characteristics[2,3]. VIIRS is thus able to detect flaming fires covering as little as 10 m^2 across much of its swath, and underground smouldering fires one to two orders of magnitude larger depending on their surface temperature.

Supplementary Fig. 2a shows the development of the fires over time and reveals a slight trend for distance from the city increasing with time, perhaps because during the early part of the fire season fires tend to be ignited closer to the city for agricultural or development purposes. This could be an influence of human activity prioritising areas close to habitation, or possibly a result of ground conditions being drier quicker close to the city where drainage channels may be better maintained. The number of active fire detections within 100 km of the city centre peaked on 13 September 2019 when 1673 individual AF hotspots were detected (1713 UTC or 0013 on 14 September local time), and this timing is also close to the peak of the $PM_{2.5}$ concentration data (Fig. 7). Supplementary Fig. 2b in fact demonstrates two main phases of fire activity around the city, the first between 13 July and 23 August and the second 30 August to 15 October, with the latter showing a far greater number of hotspots. These two phases are the cause of the two separate pulses of particularly bad air quality in the city, with their degree of agreement previously shown in Fig. 7. When the number of hotspots are normalised for area, there is a dominance of fires close to the city within the first phase (a peak of over 4000 hotspots per 100 km^2 within a 10 km radius of the city centre), compared to the second phase with a peak of only 1200 hotspots per 100 km^2. When compared to the number of hotspots in a 50 km and 100 km radius, the density of fires close to the city is disproportionately high. The area used for the CAMS 4-cell bounding box in the analysis in this paper contains 42% of the total number of hotspots within Central Kalimantan whilst containing 32% of the total FRP sum during the fire season. The same area contains 37% of the total burned area from Central Kalimantan using burned area data from Gaveau et al[4]

Supplementary Fig. 2b shows that there is good agreement in the temporal pattern of fire activity and air quality. Since the VIIRS FRP total mostly follows the pattern of the hotspot counts, the types of fire that are burning must be broadly consistent (i.e. mostly smouldering peatland fires). On the 5th to 19th August for example the FRP jumps above the hotspots counts, indicating that at this time some flaming fires may have been present. Wooster et al.[5] and Fisher et al.[6] showed how forest fires burning atop peat can generate very large amounts of $PM_{2.5}$. Also shown on Supplementary Fig. 2b is emissions estimates from the Global Fire Emissions Database (GFED5)[7] for the same spatial area. It should be noted that data from GFED is used as an input to the CAMS model, but the GFED data generally slightly precedes the CAMS data. Whilst there are some differences in the temporal pattern, there is generally agreement between the GFED data and VIIRS data.

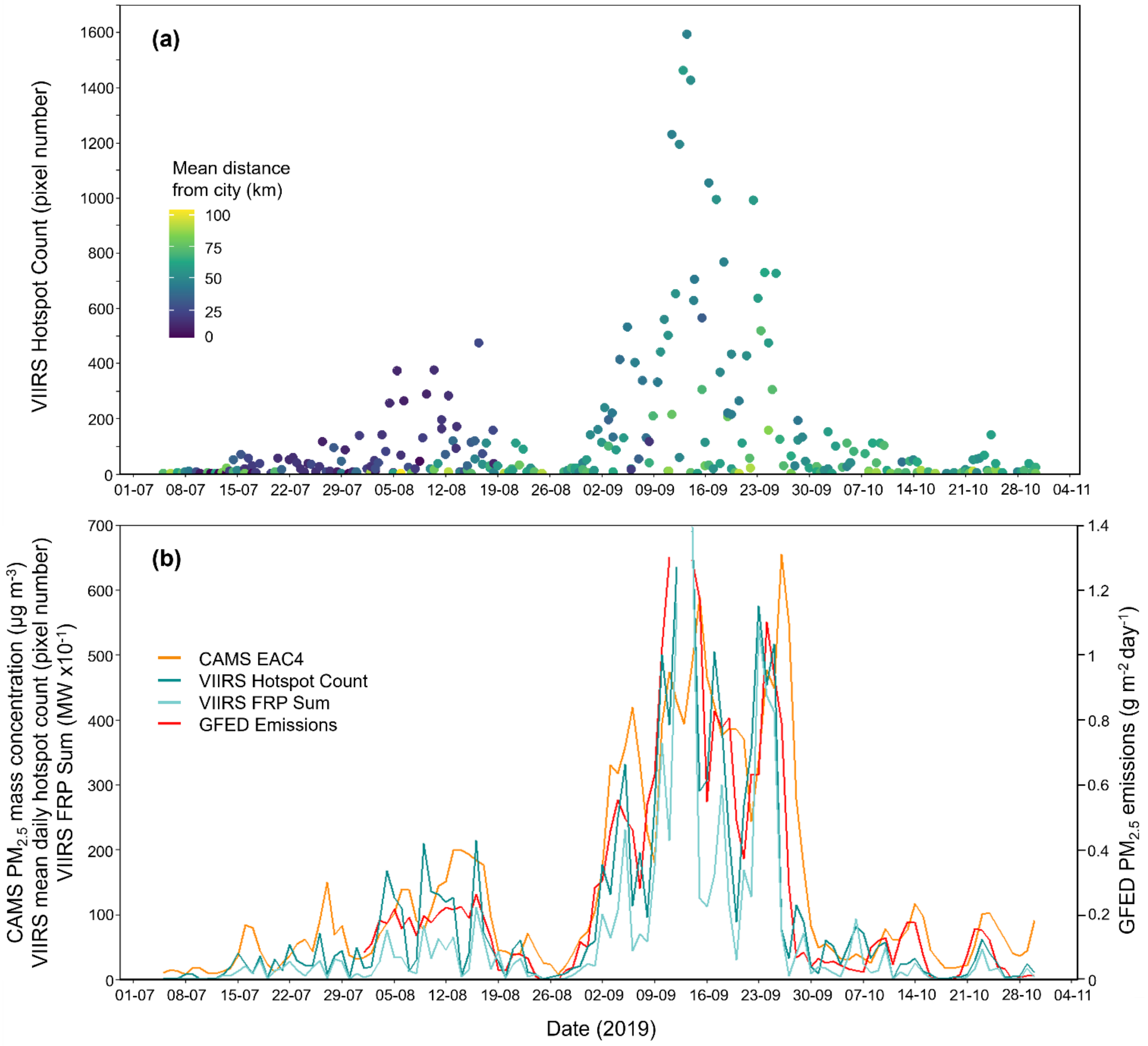

Supplementary Fig. 2 | VIIRS active fire hotspot detections near Palangka Raya. (a) Number of 375 m VIIRS active fire pixel detections for each satellite overpass (approx. 12 hours apart) within the area covered by the 4 CAMS EAC4 grid cells. Points are coloured by mean distance from the city of Palangka Raya. **(b)** Comparison of CAMS EAC4 $PM_{2.5}$ concentration with VIIRS active fire detections, and GFED $PM_{2.5}$ emissions estimates[7]. Active fire data is given in both mean daily pixel count, and FRP sum in MW.

Supplementary Notes 2: High Concentration Mass Correction for Purple Air Sensors

Due to the likelihood of airborne $PM_{2.5}$ mass concentrations in Palangka Raya being higher than the maxima suggested in the Purple Air specifications, additional laboratory testing was undertaken to characterise the sensors ability to measure such extreme concentrations. These experiments were carried out by combusting tropical peat inside a combustion chamber (2.4 x 2.4 x 2.4m) fitted with a series of small fans to ensure the smoke was well mixed. A high flow filter sampler (16.7 $l.m^{-1}$ with $PM_{2.5}$ cyclone) was run alongside 20 Purple Air sensors. PTFE filters were used in the sampler and processed identically to those used in the MiniVol sampler described in the main Methods section. Smoke concentrations were measured using 5x Plantower PMS5003 detectors (the same detectors used within the Purple Air sensors) reading at 5 second intervals across the space. A main extraction fan could be adjusted to extract smoke, whilst a secondary extract near to the smoke source could be used to limit

smoke dispersing around the smoke chamber. Fresh air could be drawn in to the chamber to balance air being removed.

Experiments were carried out at three high concentrations, based on the raw Plantower $PM_{2.5}$ readings of 1000, 1500, & 2000 µg.m^{-3} (with mean raw Purple Air values of 958, 1390, & 1777 µg.m^{-3} respectively). Variability between sensors is shown in Supplementary Fig. 3. Additional lower concentration laboratory experiments were also conducted. The filter data from the chamber experiments show that the sensors are able to make measurements up to double that provided in the PA specification, however, it is clear that there is far greater variability between individual sensors at high concentrations, and that this lower level of reliability should be factored in to any measurements made greater than 1000 µg.m^{-3}. The high concentration experiments also show that using a correction factor of 0.49 for all readings is within the range of measurements (Supplementary Fig. 4). However, when only the high concentration measurements are used, an adjustment factor of 0.67 together with an offset of -225 provides the best fit ($y=0.67x-225$, $r^2=0.99$). This adjustment factor is used on the 2.7% of datapoints from the deployment which were measured at >1000 µg.m^{-3} in order to better reflect the characteristics of the PA sensors, however it is noted that the uncertainty on these high concentrations measurements is more significant than those of the lower concentration measures.

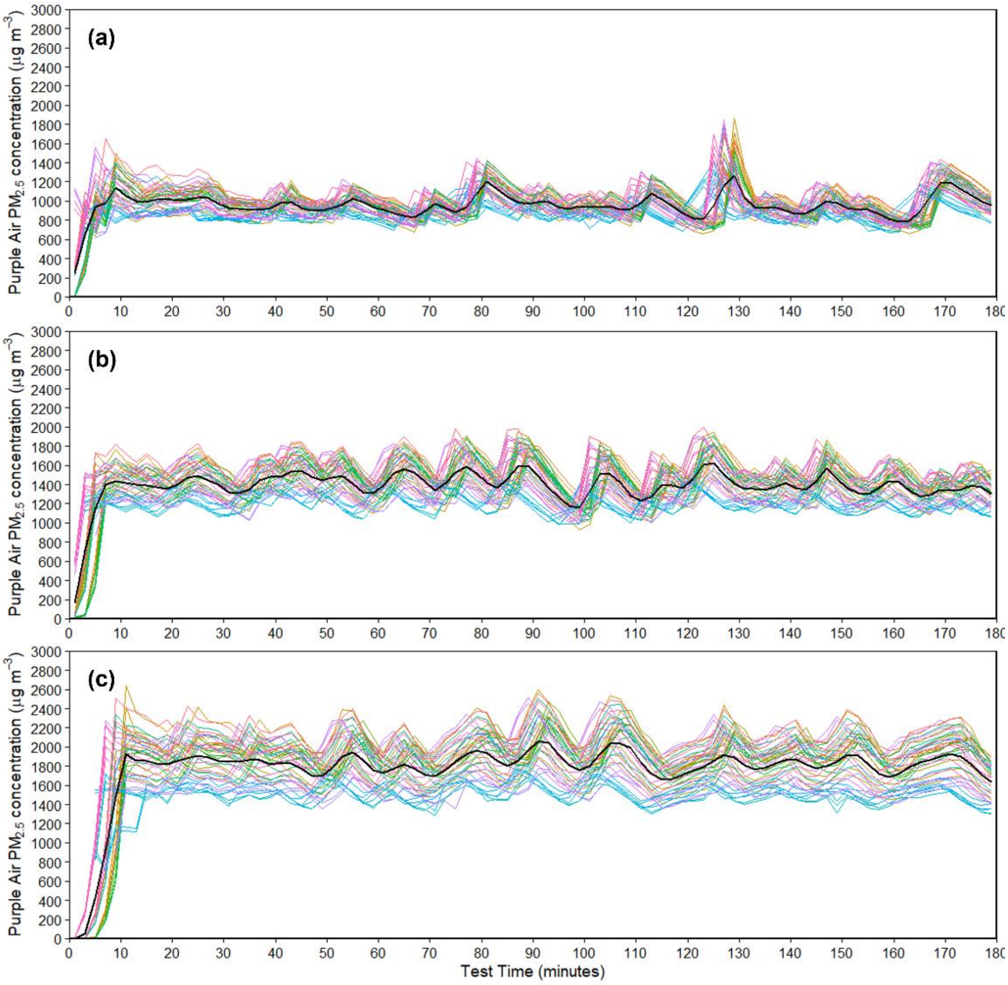

Supplementary Fig. 3 | Time series $PM_{2.5}$ mass concentration examples taken from combustion chamber tests made at target concentrations of **(a)** 1000 µg.m^{-3} **(b)** 1500 µg.m^{-3}, and **(c)** 2000 µg.m^{-3} Individual coloured lines are from individual sensors within each of the twenty Purple Air sensors used in the experiment, whilst the black line shows the mean concentration from all sensors at each 2-min timestep.

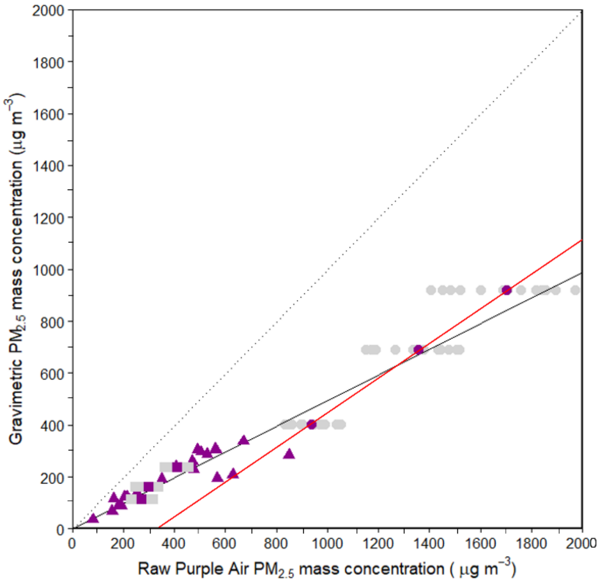

Supplementary Fig. 4 | Mass correction of Purple Air sensor $PM_{2.5}$ concentration data made against gravimetric filter samples taken using a high-volume air sampler fitted with Teflon filters (circles). The dotted line represents 1:1, the solid black line the 0.49 correction factor derived from field measurements made *in situ* in Palangka Raya (triangles), and the red line the correction factor representative of the highest concentrations derived from laboratory tests. Purple markers are the values used for the linear regression calculations and represent mean values in cases where multiple Purple Air sensors were co-located with the filter sampler. The grey circular points are the individual sensor measurements for the high concentration combustion chamber tests, and the square points are those of lower concentration also made in the same combustion chamber.

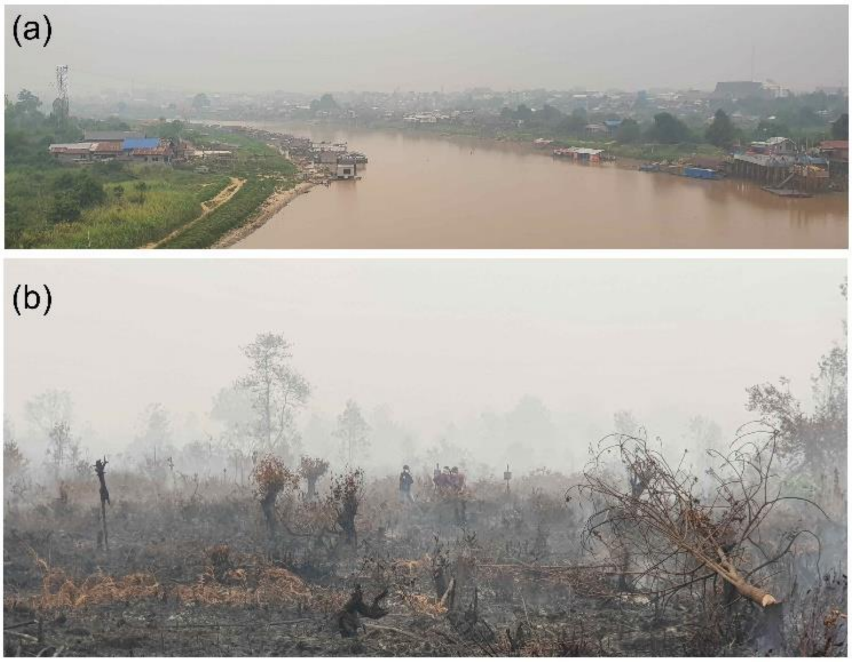

Supplementary Fig. 5 | Photographs of smoke in Palangka Raya (a) on the edge of the city and **(b)** at a fire source. Air quality conditions visibly changed in relation to distance from the fire source and type of environment even when photos were taken at similar times.

Supplementary Notes 3: Smoke Attributable Mortality Across Indonesia

Following the analysis of the Palangka Raya region, it is also possible to apply the calculations of attributable mortality from Johnston et al.[8] to other areas of Indonesia using the CAMS EAC4 Reanalysis dataset. Supplementary Table 1 provides estimates for this from Indonesia as a whole, plus Sumatra, Kalimantan, and Central Kalimantan as these areas were subject to particularly high $PM_{2.5}$ concentrations in both 2015 and 2019 fire seasons. It is notable that the background non-fire $PM_{2.5}$ concentrations are higher in Sumatra compared to Kalimantan, although the annual average $PM_{2.5}$ concentrations are similar. The smoke specific $PM_{2.5}$ concentrations calculated here show reasonable agreement with other calculation methods such as Uda et al.[9] who calculate 26 $\mu g.m^{-3}$ for Central Kalimantan in 2019, and 48$\mu g.m^{-3}$ for the same area in 2015. However, estimates by Hein et al.[10] for the mean annual increase in $PM_{2.5}$ due to peatland fires in 2015 were estimated to be 16.8 $\mu g.m^{-3}$ and 21.3 $\mu g.m^{-3}$ for Sumatra and Kalimantan respectively which is lower than estimated in this study. However, the estimated concentration for Central Kalimantan was estimated to be higher in this study compared to Hein et al.'s estimate of 33.1 $\mu g.m^{-3}$. For Indonesia as a whole, the attributable mortality calculation in this study is towards the upper end of the estimate by Koplitz et al.[11] who estimated a mean in 2015 of 91,600 (with a range of 24,000-159,200).

Area	Indonesia (whole)		Sumatra		Kalimantan		Central Kalimantan		Palangka Raya	
Year	2015	2019	2015	2019	2015	2019	2015	2019	2015	2019
Annual deaths in exposure cell	1,406,121	1,481,610	286,282	301,245	85,363	92,167	17,543	18,700	4,190	4,391
Background non-fire $PM_{2.5}$ concentration	16.82	14.58	25.61	19.02	11.52	11.43	10.19	11.12	10.83	13.03
Annual Average $PM_{2.5}$ concentration	33.17	23.78	57.96	40.92	40.34	26.95	53.92	38.49	91.87	56.56
Smoke Specific $PM_{2.5}$	16.36	9.20	32.35	21.90	28.83	15.52	43.73	27.37	81.04	43.56
Chronically affected attributable mortality	147,211	87,253	59,264	42,225	15,750	9,152	4,910	3,276	1,341	1,223

Supplementary Table 1 | Chronically affected attributable mortality calculations following the formula from Johnston et al.[8] (equation 3) for the two significant fire years of 2015 and 2019 for Indonesia as a whole, Sumatra, Kalimantan, Central Kalimantan, and Palangka Raya. Palangka Raya data is the same as is given in Table 3. The methodology for calculation of inputs to the equation are the same as those described in the main analysis and Table 3. The population data is from the 1km gridded population map[12] which is clipped to match the area of whole CAMS EAC4 reanalysis 0.75° cells, therefore the population and thus mortality values are likely an underestimate.

Mortality estimates using the Johnston et al[8] method, can also be compared to the methodology of Crippa et al[13] who separate out the proportion of mortality by key causes related to $PM_{2.5}$ exposure which are given in Supplementary Table 2. As with Crippa et al[13], the calculations use Relative Risk rates from Apte et al[14], whilst specific cause mortality rates are taken from the Global Burden of Disease Mortality Estimates for 2019[15] within the specific regions considered. Whilst the Johnston et al[8] estimates in Supplementary Table 1 are all-cause, the sums of the specific causes in Supplementary Table 2 show a broad similarity. It should be noted that for both sets of calculations the same exposure estimates are used (from the CAMS EAC4 Reanalysis dataset), but the relative risk component of the equation uses different inputs.

Area	Indonesia (whole)		Sumatra		Kalimantan		Central Kalimantan		Palangka Raya	
Year	2015	2019	2015	2019	2015	2019	2015	2019	2015	2019
Chronic obstructive pulmonary disease	8,468	5,632	2,930	2,379	985	650	289	209	108	68
Lung cancer	5,315	3,455	1,965	1,554	658	424	202	144	78	48
Lower respiratory infection	11,177	6,241	5,259	3,620	1,382	764	489	316	214	123
Ischemic heart disease (aged >25)	55,122	40,320	13,713	13,006	5,883	4,258	2,176	1,689	671	477
Ischemic stroke (ages >25)	69,966	44,104	20,231	18,397	7,410	4,732	1,872	1,418	588	444
Sum of disease specific mortality	150,048	99,753	44,097	38,956	16,317	10,828	5,029	3,776	1,659	1,158

Supplementary Table 2 | Attributable mortality calculations following the methodology of Crippa et al[13] for 2015 and 2019 for Indonesia as a whole, Sumatra, Kalimantan, Central Kalimantan, and Palangka Raya. The population data is from the 1km gridded population map[12] which is clipped to match the area of whole CAMS EAC4 reanalysis 0.75° cells.

Supplementary References

1. Innes, A. *et al.* The CAMS reanalysis of atmospheric composition, *Atmospheric Chemistry and Physics*, **19**, 2515-2556 (2019).
2. Schroeder, W., Oliva, P., Giglio, L., Csiszar, I.A. The new VIIRS 375 m active fire detection data product: Algorithm description and initial assessment, *Remote Sensing of Environment*, **143**, 85-96 (2014).
3. Zhang, X., Liu, L., Yan, D. Comparisons of global land surface seasonality and phenology derived from AVHRR, MODIS, and VIIRS data, *Journal of Geophysical Research: Biogeosciences*, **122**, 1506-1525 (2017).
4. Gaveau, D.K.A., Descals, A., Salim, M.A., Sheil, D., Sloan, S. Refined burned-area mapping protocol using Sentinel-2 data increases estimate of 2019 Indonesian burning, Earth Systems Science Data, 13, 5353-5368 (2021).
5. Wooster, M.J. *et al*. New Tropical Peatland Gas and Particulate Emissions Factors Indicate 2015 Indonesian Fires Released Far More Particulate Matter (but Less Methane) than Current Inventories Imply, *Remote Sensing*, **10**, 495 (2018).
6. Fisher, D., Wooster, M.J., Xu, W., Thomas, G. and Lestari, P. Top-down estimation of particulate matter emissions from extreme tropical peatland fires using geostationary satellite fire radiative power observations. *Sensors*, **20**(24), p.7075 (2020).
7. Chen, Y.*et al*. Multi-decadal trends and variability in burned area from the fifth version of the Global Fire Emissions Database (GFED5), Earth System Science Data, 15, 5227–5259, (2023)
8. Johnston, F. H. *et al.* Estimated Global Mortality Attributable to Smoke from Landscape Fires, *Environmental Health Perspectives*, **120**(5), 695-701 (2012).
9. Uda, S.K., Hein, L., Atmoko, D. Assessing the health impacts of peatland fires: a case study for Central Kalimantan, Indonesia, *Environmental Science and Pollution Research*, **26**, 31315-31327 (2019).
10. Hein, L. *et al*. The health impacts of Indonesian peatland fires. *Environmental Health*, **21**(1), 62 (2022).
11. Koplitz, S.N. *et al*. Public health impacts of the severe haze in Equatorial Asia in September-October 2015: demonstration of a new framework for informing fire management strategies to reduce downwind smoke exposure, *Environmental Research Letters*, **11**, 094023 (2016).
12. Tatem, A.J. WorldPop, open data for spatial demography, *Scientific Data*, **4**:170004 (2017).
13. Crippa, P. *et al*. Population exposure to hazardous air quality due to the 2015 fires in Equatorial Asia, *Scientific Reports*, **6**:37074 (2016).
14. Apte, J.S., Marshall, J.D., Cohen, A.J., Brauer, M. Addressing Global Mortality from Ambient $PM_{2.5}$, *Environmental Science & Technology*, **49**, 8057-8066 (2015).
15. Institute for Health Metrics and Evaluation (IHME) Global Burden of Disease Study 2021 (GBD 2021) Cause-Specific Mortality 1990-2021. Seattle, United States of America: Institute for Health Metrics and Evaluation (IHME) (2024).
